# Natural inhibitors: A sustainable way to combat aflatoxins

**DOI:** 10.3389/fmicb.2022.993834

**Published:** 2022-12-08

**Authors:** Malik M. Ahmad, Firdaus Qamar, Monica Saifi, Malik Zainul Abdin

**Affiliations:** ^1^Department of Agriculture, Integral Institute of Agricultural Science and Technology (IIAST), Integral University, Lucknow, India; ^2^CTPD, Department of Biotechnology, School of Chemical and Life Sciences, New Delhi, India

**Keywords:** inhibitors, natural, sustainable, mycotoxins, aflatoxins

## Abstract

Among a few hundred mycotoxins, aflatoxins had always posed a major threat to the world. Apart from *A. flavus*, *A. parasiticus*, and *A. nomius* of *Aspergillus* genus, which are most toxin-producing strains, several fungal bodies including *Fusarium*, *Penicillium*, and *Alternaria* that can biosynthesis aflatoxins. Basically, there are four different types of aflatoxins (Aflatoxin B1 (AFB1), Aflatoxin B2 (AFB2), Aflatoxin G1 (AFG1), Aflatoxin G2 (AFG2)) are produced as secondary metabolites. There are certainly other types of aflatoxins found but they are the by-products of these toxins. The fungal agents generally infect the food crops during harvesting, storing, and/or transporting; making a heavy post-harvest as well as economic loss in both developed and developing countries. And while ingesting the crop products, these toxins get into the dietary system causing aflatoxicosis, liver cirrhosis, etc. Therefore, it is imperative to search for certain ways to control the spread of infections and/or production of these toxins which may also not harm the crop harvest. In this review, we are going to discuss some sustainable methods that can effectively control the spread of infection and inhibit the biosynthesis of aflatoxins.

## Introduction

Fungi are minute organisms that dwell in soil and air and are often associated with food spoilage and biodeterioration. After fulfilling its primary needs, a toxigenic fungus produces some sort of secondary metabolites like mycotoxins which can contaminate the food and feed products that can cause food allergies. These mycotoxins are thought to provide a competitive edge over the non-producers (Abdin et al., 2010). Mycotoxin contamination of different cereal, pulse, and oilseed crops is a universal management issue for both government and agriculturalists. These toxins jeopardize the food and feed safety, as well as the agro-based economy and crop-dependent small-scale companies (Kumar et al., 2021). The principal mycotoxin-producing fungus in nature is *Aspergillus, Penicillium*, and *Fusarium*; however, only a few species within these genera are capable of toxin production such as aflatoxins, cyclopiazonic acid, ochratoxin A, patulin, fumonisins, trichothecenes or zearalenone, etc.

A class of structurally related chemicals known as aflatoxins (AFs), a toxin-producing polyketide, is present in a wide range of food and feed crops around the world, particularly maize, peanuts, tree nuts, and oilseeds (Abdin et al., 2010). AFs are one of the mycotoxins produced by certain *Aspergillus* species during secondary metabolism. It has been labeled as a carcinogen, a biological weapon, and an immunosuppressant. Only *A. flavus, A. parasiticus*, and *A. nomius* have been well identified as aflatoxin producers among the species listed in the section *Flavi.* The most important toxin-producing species in the group are *A. flavus* and *A. parasiticus*. Although both these species can deposit mycotoxins in food, the poisons they create are somewhat different. The majority of *A. flavus* isolates are found to secrete B1 and B2 aflatoxins and cyclopiazonic acid (CPA), but certain strains of *A. flavus* had been found to produce G1 and G2 type of aflatoxins as well as CPA (Geiser et al., 2000; Cardwell and Cotty, 2002). *A. parasiticus*, on the other hand, produces all four types of aflatoxins but not CPA. Each of these mycotoxins has the potential to negatively affect the humans if consumed in a significant amount. Aflatoxins B1 and B2 have been associated with cirrhosis and acute liver injury and have been classified as oncogenic, teratogenic, and immunosuppressive. The aflatoxins G1 and G2 have similar effects, and aflatoxin G1 is only slightly less lethal than aflatoxin B1. Aflatoxin M1 and M2 can be found in dairy products from animals given aflatoxin-contaminated feed. CPA has also been found in agricultural products, and in lab animals, it has been linked to gastrointestinal and neurological diseases (Bryden, 1991; Bhatnagar et al., 2008). AFB1, the most toxigenic of all the AFs, is thought to be present in nearly a quarter of the world’s food supply. As a result, 20 ppb has been established as the maximum permissible level for total AFs in foods fit for human consumption by the Food and Drug Administration (FDA) in the United States and regulatory agencies in many other nations (FDA (Food and Drug Administration), 2011).

Extensive research has been conducted to efficiently control and manage AF contamination in crops to assure global food and feed supply safety. Although, to address the issue of postharvest fungal diseases, a variety of physical and chemical treatments are used. But each of these methods has its own set of constraints (Sonker et al., 2015). Due to the careless approach and excessive pesticide use, new secondary pests have evolved in the storage of food commodities, which has been linked to fungal resistance (da Cruz Cabral et al., 2013). This abuse of pesticides has led to the rise in of dangerous fungicide residues on food and the environment (Sonker et al., 2015). In this regard, the ability of plants, bacteria, microalgae, fungi, and actinomycetes to inhibit the growth of toxic fungi and the formation of AF has been studied. Significant attempts have been undertaken to identify organisms that can hinder AF production through co-culturing techniques with hopes of uncovering bio-control agents and novel antagonistic metabolites. Numerous molecular studies of AF had been conducted aiming out how plants and microorganisms, as well as their bioactive metabolites, influence the formation of AF (Razzaghi-Abyaneh et al., 2011). We describe here some AF inhibitors that can either be a molecule, a plant, or a microorganism, with a focus on their putative cellular and molecular mechanisms of action.

## Microorganisms and their metabolite-based inhibitions

Antifungal chemicals have been discovered in fungi, bacteria, and actinomycetes of various species. Aflatoxin under the field circumstances is decreased using bio-pesticides derived from atoxigenic strains. Ren et al. (2020) reported that around 61% of articles published included bacterial antagonists, mainly *Bacillus*, for managing aflatoxin production followed using antagonistic fungi (27%; majorly atoxigenic *Aspergillus* spp.) or yeast (12%). Numerous bacterial and actinomycete species, including *Ralstonia, Burkholderia, Pseudomonas, Bacillus, Lactobacilli, Stenotrophomonas,* and *Streptomyces* have successfully prevented the *in vitro* aflatoxin production by limiting the development of *Aspergillus* species. In terrestrial actinomycetes, particularly those belonging to the genus *Streptomyces*, antifungal and AF inhibitory metabolites are produced in abundance (Deshpande et al., 1988). In terms of AF regulation, mycoviruses and RNA silencing have also gotten a lot of attention (Hammond et al., 2008; Schmidt, 2009).

## Bacteria and actinomycetes

Kimura and Hirano (1988) developed a *Bacillus subtilis* (NK-330) strain that suppressed *Aspergillus flavus* and *Aspergillus parasiticus* growth and aflatoxin formation. In an *in vitro* study utilizing inoculated cotton seed, six of 892 bacterial isolates native to cotton reduced *Aspergillus flavus* growth (Misaghi et al., 1995). Darsanaki and Miri (2013) in their study found that only *Flavobacterium aurantiacum* B-184 was found to be effective for the degradation of aflatoxins while Shu et al. (2018) identified *Bacillus velezensis* DY3108 strain supernatant can also be effective. In a study, *Bacillus megaterium* and *Bacillus subtilis* stopped the aflatoxin production in *A. flavus* and *A. parasiticus* by 100%, respectively (Kong et al., 2014; Siahmoshteh et al., 2018). During an experiment, Pereyra et al., 2018 showed that *B. mojavensis* RC1A reduced the AFB1 production while *B. subtilis* RC6A inhibited the fungal growth by producing fengycines, surfactins, iturins and bacillomycins types of antifungal lipopeptides. It was assumed that these potential inhibitors are like AFLS size and thus form a dysfunctional AFLS/AFLR activation complex that can possibly bind with aflatoxin pathway-structural genes, consequently inhibiting or terminating the transcription process (Kong et al., 2014). Some other *Bacillus* species like *B. amyloliquefaciens, B. cereus, B. mycoides,* and *B. pumilus* have also been found to be the most effective against inhibiting the aflatoxin production.

Using the antagonistic strains of *Ralstonia, Lactobacilli, Burkholderia* and *Pseudomonas* sp. reduces the *A. flavus* growth significantly (Akocak et al., 2015; Yang et al., 2017). When *Pseudomonas cepacia* (Dl) was injected alongside *A. flavus* in field tests, the bacterium greatly reduced *A. flavus* damage to locules by 41–100% (Misaghi et al., 1995). Yang et al. (2017) investigated *Pseudomonas fluorescens* strain 3JW1, a broad-spectrum antimicrobial bacterium, stopped the production of AFB1 in PDB and peanut medium by 97.8 and 99.4%, respectively and reduced the aflatoxin B1 production by 55.8% as soon as the same bacterium was applied onto the peanut kernels. The aflatoxins produced by *A. flavus* on rice grains were significantly reduced to 8.68% by *P. protegens* AS15 with an 82.9% reduction in aflatoxin compared to control (Mannaa et al., 2017). The gram-negative bacillus *Achromobacter xylosoxidans* belongs to the *Alcaligenaceae* family and is non-fermentative which had a biological activity that prevented *A. parasiticus* from producing AF (Yan et al., 2004). *Lactobacillus* are lactic acid bacteria that ferment sugars to produce lactic acid and are most used microorganism in food industries. Many researchers found that *L. plantarum* is the most effective lactobacillus species that can act as biocontrol agent out of *L. delbrueckii, subsp. lactis, L. reuteri, L. plantarum, L. acidophilus, L. paraplantarum, L. rhamnosus, L. fermentum,* and *L. pentosus* (Ahlberg et al., 2017; Ghanbari et al., 2018).

Aflatoxin generation by *Aspergillus flavus* was suppressed *in vitro* by saprophytic yeasts isolated from almond, pistachio, and walnut fruits (Hua et al., 1999). In a Petri dish assay, a *Candida krusei* strain and a *Pichia anomala* strain reduced aflatoxin formation by 96 and 99%, respectively. A chemical has been found in an ascomycete (*Rosellinia necatrix*), a marine sponge (*Rhaphisia pallida*), and *Halobacillus litoralis*, a marine bacterium (Yan et al., 2004). Using a tip culture approach, the compound’s IC_50_ value for AFB1 production was ascertained to be 200 g/mL. At a high concentration of 6,000 g/mL, it inhibited *A. parasiticus* growth. Takeuchi et al. (1991) discovered dioctatin A from *Streptomyces* sp. SA-2581 is something of a suppressor of human dipeptidyl peptidase II, which explains the compound’s immunosuppressive properties. Dioctatin A is likewise a powerful inhibitor of AF synthesis by *A. parasiticus*, according to Yoshinari et al. (2007). They discovered that an IC_50_ value of 4.0 μM suppressed AF synthesis without affecting fungal growth. Dioctatin A has some known advantages, including a straightforward structure, a lack of toxicity for mammals, a reduction in AF forming in a model contagion system on raw peanuts, suppression of AF as well as conidiogenesis without impacting fungal infections (reducing the likelihood of resistance propagation), and a focus on secondary metabolism rather than primary metabolism.

Aflastatin A was initially isolated from solvent extracts of soil isolation of *Streptomyces sp*. MRI142’s mycelial cake. It completely reduced AF production by *A. parasiticus* NRRL2999 at a dose of 0.5 g/mL without affecting fungal growth in solid and liquid cultures (Ono et al., 1997). The influence on the AF biosynthesis pathway and glucose metabolism in *A. parasiticus* was used to investigate its inhibitory mechanism (Kondo et al., 2001). *Streptomyces* species-specific changes in the aflR/aflS ratio appeared to inhibit aflM and aflS (Verheecke et al., 2015) and thus by changing the transcription of the aflR gene, aflastatin A can directly limit the production of AF, or it can do so in a more indirect way by interfering with the carbon metabolism regulatory system (Kondo et al., 2001).

Blasticidin A, a peptidyl nucleoside antibiotic, isolated from *Streptomyces griseochromogenes* was first described as an anti-phytopathogenic agent by Fukunaga et al. (1955). It is a potent inhibitor of the AF biosynthesis of *A. parasiticus* (Sakuda et al., 2000). Based on two-dimensional differential gel electrophoresis (2D-DIGE), Yoshinari et al. (2010) discovered that blasticidin A decreased AF (total of B1 and G1) synthesis and fungal growth with IC_50_ values of 0.25 and 1.6 μM, respectively. Protein synthesis was found to be the potential target location for blasticidin A in toxic fungi. In the same experiment, it was shown that blasticidin S, a different peptidyl nucleoside antibiotic, had a somewhat inhibitive effect on fungal growth (IC_50_ > 1,000 μM) and was an inhibitor of AF production (IC_50_ = 28 μM).

## Mushrooms and microfungi

Mushrooms have been researched as potential AF contamination control agents because of their hepatoprotective effects (Reverberi et al., 2005; Zjalic et al., 2006). Reverberi et al. (2005) found that pure β-glucans from the edible basidiomycetous fungus *Lentinula edodes* and its culture filtrate significantly suppressed AF synthesis by *A. parasiticus*. They hypothesized that the reduction of AF formation by β-glucans and *L. edodes* culture filtrate was due to a stimulation of the fungal anti-oxidant system, which activates antioxidant enzymes like SOD, catalase, and glutathione peroxidase. As a result, a delay in AF gene transcription causes a significant reduction in AF biosynthesis. The culture filtrate of *L. edodes* is a promising method for controlling AF contamination of crops before and after harvest. Zjalic et al. (2006) tested *Trametes versicolor*, an industrially important fungus, for antifungal efficacy against an AF-producing *A. parasiticus*. *T. versicolor* culture filtrate’s antioxidant effect may be linked to β-glucan, a free radical scavenging agent that inhibits AF production. *T. versicolor* filtrate also dramatically decreased norA expression and markedly delayed aflR transcription, according to RT-PCR investigations of AF biosynthesis genes. Lee et al. (2007) found that at a concentration of 1 M, wortmannin inhibited fungal growth, asexual sporulation, the production of AF, and the expression of AF pathway genes ver1 and nor1. Similar to the signaling mechanism discovered by Rondinone et al. (2000) for human hepatocytes, the decrease in AF synthesis appears to interfere with this pathway, which is regulated by phosphatidylinositol 3-kinase.

## Plant-based inhibitions

Our knowledge of AF-producing fungi’s survival in nature and just how they assault host plants to produce AF has improved with the use of genomes, proteomics, and metabolomics data (Rajasekaran et al., 2006; Kim et al., 2007; Bhatnagar et al., 2008; Brown et al., 2010). In response to fungal invasion and infection, plants produce enzymes that break down fungal cell walls, specific inhibitors of fungal growth and/or AF synthesis, proteins that are sensitive to ROS and stress, increased lignification and cell wall cross-linking, and host cell death at the sites of infection (Liang et al., 2006; Bhatnagar et al., 2008). Plant-based metabolites are a possible alternative, as plants produce a wide range of chemicals as part of their natural development or in reaction to stress or infections. There are several plant-derived inhibitors of aflatoxin production (Bhatnagar and McCormick, 1988). The search for new inhibitors of AF biosynthesis in natural sources has been intense, with many bioactive AF inhibitory chemicals such as terpenes, phenolics, phenylpropanoids, and nitrogen-containing compounds having been found in herbal treatments (Razzaghi-Abyaneh et al., 2009). The strongest antifungal and AFB1 inhibitory activity were seen in chloroformic extracts of *Albizia amara*, *Cassia spectabilis*, and *Solanum indicum*, as well as methanolic extracts of *Acacia catechu, Albizia saman*, and *Anogeissus latifolia* (Thippeswamy et al., 2014). Certain plant-derived volatile chemicals are examples of natural products that may have the ability to improve host plant tolerance against *Aspergillus flavus* infection. Aflatoxin production and the fungal growth are both altered when cotton-leaf or maize volatiles are present during *in vitro* culture of aflatoxigenic strains of *A. flavus* and *A. parasiticus* (Zeringue Jr, 1992; Zeringue et al., 1996). Specific volatile chemicals have been studied not just for their effect on fungal growth and toxin generation, but also for their effect on fungal morphology (Greene-McDowelle et al., 1999; Wright et al., 2000). In another study, Wright et al. (2000) looked at how three volatile compounds, *viz.*, n-decyl aldehyde, hexanol, and octanol, generated from corn affected *A. parasiticus*. These three chemicals were previously discovered to be expressed in a variety of ways in five *Aspergillus*-resistant maize variants and three susceptible maize varieties. In contrast to octanol, only n-decyl aldehyde inhibited the manufacture of aflatoxin in *A. parasiticus*. While n-decyl aldehyde had less impact on radial growth than other volatiles, yet it did cause a greater proportion of distinctly aerial hyphae and significantly fewer conidiophores in the treated colonies than in the reference and other aldehyde intervention groups. Several other chemicals found in corn, peanuts, and walnuts have a substantial impact on aflatoxin production. For example, aflatoxin formation was inhibited by 4-acetyl-benzoxazolin-2-one (ABOA), anthocyanins, and similar flavonoids and carotenoids with an α-ionone type ring (from maize) (Miller et al., 1996; Norton, 1997, 1999). At concentrations of 50 μg/mL, β-carotenes from maize were more effective, almost 90%, than those of peanuts in reducing aflatoxin formation (Wicklow et al., 1998). *Aspergillus flavus* spore germination and growth, as well as aflatoxin biosynthesis, were found to be inhibited by naphthoquinones present in walnut husks (Mahoney et al., 2000). Several natural substances (anthraquinones, coumarins, and llavone-type flavonoids) derived from a variety of plants have been demonstrated to be effective inhibitors of aflatoxin B1-8,9-epoxide production (Lee et al., 2001).

Monoterpenes, flavonoids, furanocoumarins, and phenylpropanoids of both *Anethum graveolens* and *Petroselinum crispum* (Apiaceae family), have been found to be related to a variety of biological roles (Crowden et al., 1969). Phenylpropanoids are a type of plant phenol with a three-carbon side chain and a phenyl ring which is made from phenylalanine *via* shikimic acid pathway (Korkina, 2007). Plant-derived phenolics such as flavonoids, coumarins, and lignins are produced through the metabolism of phenylpropanoids (MacRae and Towers, 1984). Dillapiol, with an IC_50_ of 0.15 M and no impact on fungal growth or AFB1 biosynthesis, a phenylpropanoid molecule extracted from *A. graveolens* leaf as essential oil was shown to be a potent inhibitor of AFG1 production by *A. parasiticus* (Razzaghi-Abyaneh et al., 2007). In the same investigation, apiol, a phenylpropanoid extracted from the seed essential oil of *P. crispum*, had similar effect to dillapiol, with an IC_50_ value of 0.24 M for AFG1. These phenylpropanoids are thought to block AFG1 production by inhibiting CypA, a cytochrome P_450_-dependent monooxygenase involved in the AF biosynthetic pathway’s conversion of *O*-methyl sterigmatocystin to AFG1.

One of the most widely used medicinal plants in the world is *Matricaria recutita* L., a member of the Asteraceae family (Salamon, 1992). The major ingredient is α-bisabolol, a stable natural monocyclic sesquiterpene alcohol (Tolouee et al., 2010). This plant is generally considered safe by the FDA (GRAS) (Bradley, 1992; Newall et al., 1996). Yoshinari et al. (2008) used a microbioassay technique to screen 110 commercial essential oils from various plants and discovered a unique biological activity from *M. recutita* had suppressed AFG1 synthesis in *A. parasiticus* NRRL2999 at a dose of 100 g/mL. Spiroethers may reduce AFG1 synthesis by inhibiting the activity of a cytochrome P_450_-dependent enzyme. The CypA, a P_450_ monooxygenase enzyme, is most likely the target of spiroethers inhibition on AFG1 production.

Another Asteraceae family member, *Ageratum conyzoides*, is an exotic plant with exceptional levels of environmental adaptability. The primary chemical components of this plant have been linked to many health advantages, including terpenoids, flavonoids, and phenolics, which have antibacterial activities against various bacteria and fungi (Kong et al., 2004). According to Nogueira et al. (2010), the essential oil of the plant’s aerial parts (0.1 g/mL) fully prevented *A. flavus* IMI190 from producing AFB1, as well as retarded the growth of the fungus. Two dimethyl chromenes, precocenes I and II, were found to be the primary ingredients of *A. conyzoides* essential oil. Ultrastructural alterations in the plasma membrane and membranous organelles, particularly the mitochondria, were discovered using electron microscopy on oil-treated fungal formations. At concentrations of more than 10% (v/v), aqueous extracts of *Azadirachta indica* leaves and seeds limit AF synthesis by *A. parasiticus* without affecting the fungal growth. Razzaghi-Abyaneh et al. (2005) found that the cultures with 50% neem leaf and seed extracts suppressed AF formation by 90 and 65%, respectively, after 96 h. The integrity of cell barriers, especially the cell wall, is crucial in the control of AF biosynthesis and excretion, according to electron microscope analysis of treated and untreated fungi. This relationship was found between decreased AF production and morphological changes.

All 11 of the carotenoids studied by Norton (1997) in his investigation for the impact of maize carotenoids on *A. flavus* AF production were examined, except for α-tocopherol, which significantly reduced AFB1 production. The carotenoids with the α-ionone ring, such as α-carotene, lutein, and α-ionone, were the most potent suppressing AFB1 synthesis by more than 90%. There is evidence that α-carotene has a target site(s) in the AF pathway before NOR biosynthesis. Based on the structure/activity data, two explanations for the observed suppression were put forth: modification of cell membranes, which directly impacts cytosolic polyketide synthase, and specific contact with hydrophobic domains of AF pathway enzymes.

## Phenolic-based inhibitions

The most prevalent secondary metabolites in plants are phenolics, which have over 8,000 distinct structural variations, ranging from straightforward phenolic acids to more intricate structures like tannins (Dai and Mumper, 2010). Numerous studies have examined how phenolics affect AF synthesis and microbial development (Hua et al., 1999; Kim et al., 2005, 2006; Razzaghi-Abyaneh et al., 2008). Zhou et al. (2015) tried tea-derived polyphenol combinations and several individual polyphenols for observing inhibition of both *A. flavus* fungi as well as aflatoxin. It was observed that only quercetin inhibited the fungal growth at 800 μg/mL while catechin mixtures and polyphenols at individual levels differentially obstructed the fungal AFB1 biosynthesis. Gomaa and El Nour (2014) discovered that green tea phenolic extract was utilized as it prevented the generation of aflatoxin while having no impact on fungal mycelial proliferation. The innovative biological activity of *Satureja hortensis* L. leaf essential oil as a potent inhibitor of *A. parasiticus* NRRL2999 AF production was discovered by Razzaghi-Abyaneh et al. (2008). These phenolics inhibited AFB1 and AFG1 synthesis in a dose-dependent manner at doses ranging from 0.041 to 1.32 mM in all two-fold dilutions. The compounds’ IC_50_ values for AFB1 and AFG1 were 0.50 and 0.06 mM, respectively. Because these phenolics are powerful antioxidants, their inhibitory effects on AF synthesis are most likely mediated through the fungus’ oxidative stress levels. Using an *S. cerevisiae* model system, Kim et al. (2006) showed that natural phenolics effectively work in conjunction with well-known antifungal medications such as carboxin and strobilurin. They discovered that the phenolics salicylic acid, vanillin, thymol, vanillyl acetone, and cinnamic acid suppress *A. flavus* growth by targeting the mitochondrial oxidative stress defense mechanism. Because mitochondria provide acetyl-CoA, a crucial component for the production of AF, antifungal phenolics’ inhibitory effects on AF production may be due in part to disruption of the mitochondrial respiration chain.

## Antioxidant-based inhibitions

Given that the polyketide synthase initiates aflatoxin production and that its precursors are highly oxygenated, it has been speculated that aflatoxin biosynthesis may represent a method fungal of defense mechanism against oxidative stress (Kim et al., 2005). However, it has been demonstrated that certain antioxidants prevent the formation of aflatoxin. Most of the natural antioxidants are found in plants materials like polyphenols (phenolic acids, flavonoids, anthocyanins, lignans and stilbenes), carotenoids (xanthophylls and carotenes) and vitamins (vitamin E and C) (Baiano and del Nobile, 2015). Aflatoxin producing fungi infects plants and produce aflatoxins but plants also produce antioxidants thus, they may impede AF biosynthesis by influencing toxigenic fungi’s oxidative stress responses. Zhao et al. (2018) observed that by altering the transcription factors FarB and CreA, the antioxidant gallic acid prevents *Aspergillus flavus* from producing aflatoxins. In an RNA-based study, Ren et al. (2020) found that 3.5% of ethanol hinders the aflatoxin B1 production in *Aspergillus flavus*. Specifically, ethanol exposure resulted in the downregulation of the stress-related transcription factor srrA and the upregulation of the bZIP transcription factor ap-1, the C2H2 transcription factors msnA and mtfA, as well as several genes of anti-oxidant enzymes, such as Cat, Cat1, Cat2, CatA, and the Cu, Zn superoxide dismutase gene sod1. Gomaa and El Nour (2014) discovered that cultures enriched with gallic acid (a potent antioxidant) and an extract rich in phenolic compounds from green tea had no detectable aflatoxins at all. Caffeic acid is also a well-known antioxidant secondary metabolite found in many naturally occurring plant secondary metabolites. Caffeic acid is a critical step in the formation of lignin and is found in all plants. Kim et al. (2008) state that caffeic acid has been used as a marker to clarify the antioxidant-based regulation of AF biosynthesis. Caffeic acid was added to fat-riched growth media at a final concentration of 12 mM, which inhibited AF synthesis by *A. flavus* NRRL3357 by >95% while not affecting fungal growth. A series of events underlying the caffeic acid-induced suppression of AF synthesis by *A. flavus* was proposed by microarray analysis of a large number of genes engaged in lipid metabolic pathways, cell wall integrity as well as transport, and oxidative/antioxidative exercises.

## Essential oil-based inhibitions

Fumigation with ginger *Zingiber officinale Roscoe* essential oil (GEO) has strong antifungal efficacy against *A. flavus*, according to Nerilo et al. (2020). It has been demonstrated *in situ* (maize grains) in a dose-dependent way. At doses of 25 and 50 g/g, the GEO prevented aflatoxin synthesis while also controlling fungal growth. As a result, GEO can be utilized as a successful and non-toxic alternative to traditional therapies for the natural control of *A. flavus* in stored maize grains. *Citronella* EO at 1000 g/g reduced *A. flavus* growth for just 3 days, but citronella EO at 5000 g/g completely inhibited *A. flavus* growth for 28 days, according to Thanaboripat et al. (2004). After 300 h of incubation, Boldo essential oil dosages of more than 1.5 g/g were shown to suppress *A. flavus* by 93–100% at A_w_ of 0.98, 0.95, and 0.93 by Passone et al. (2013). The ability to maintain antifungal activity was only seen at the 3,000 g/g of the highest concentration of the five EOs tested, which included *Lippia maizeeto* var. *Integrifolia* (Griseb), *Syzygium aromaticum* L. (Clove), *Pneumus boldus* Mol. (Boldo), *Hedeoma multiflora* Benth (Mountain thyme), and *Pimpinella anisum* L. (Anisseed) (Bluma and Etcheverry, 2008). In a 6-month *in vivo* investigation, the *Rosmarinus officinalis* EO at 1500 g/g substantially protected *Piper nigrum* fruits against *A. flavus* (Prakash et al., 2015). With 300 g/g of *Artemisia nilagirica* EO, table grapes had a nine-day shelf life against *A. flavus*, *A. niger*, and *A. ochraceus* (Sonker et al., 2015).

In 1820, German scientist Friedlieb Ferdinand Runge found the xanthine alkaloid caffeine in coffee. In submerged cultures containing 2 mg/mL caffeine, Buchanan and Lewis (1984) observed practically full inhibition of AF synthesis as well as a significant suppression (80–90%) of *A. parasiticus* growth. Based on findings from feeding experiments with [U-C14] glucose and enzymatic assays, Buchanan and Levis hypothesized that caffeine reduces AF production by interfering with fungal cells’ respiratory systems and preventing glucose uptake, which is necessary for the production of acetyl-CoA, the compound that makes up AFs. Salicylaldehyde reduces AF synthesis by 13–45% in *A. flavus* and *A. parasiticus* at a dose of 9.5 mM and retards fungal development, according to Kim et al. (2011). Using the model of yeast gene deletion mutants, they argued that salicylaldehyde targets the fungal antioxidant system and that vacuolar detoxification plays a major role in fungal resilience. Maize kernel resistance-related proteins (RAPs) have been discovered as favorable breeding markers in comparative proteomics investigations (Bhatnagar et al., 2008; Brown et al., 2010). Antifungal, storage, and stress-responsive proteins were the three principal categories of RAPs. The 14 kDa trypsin inhibitor, a RAP from maize kernel, has been used to demonstrate resistance to AF contamination and fungal invasion (Brown et al., 2010). By inhibiting *A. flavus*, α-amylase, a fungal pathogenic factor, this trypsin inhibitor indirectly inhibits AF synthesis (Chen et al., 1998; Fakhoury and Woloshuk, 1999). Alpha-amylase is one of the extracellular hydrolases in *A. flavus* that breaks down starch into glucose and maltose, both of which are important for fungus growth.

## Amino acid-based inhibitions

One of the many factors known to influence aflatoxin biosynthesis is amino acid catabolism. During aflatoxin biosynthesis, *A. flavus* readily absorbed the amino acids methionine, phenylalanine, tyrosine, tryptophan, and acetate (Adye and Mateles, 1964). Proline and asparagine were discovered to promote aflatoxin formation in both *A. flavus* and *A. parasiticus* in addition to being easily integrated into aflatoxin (Payne and Hagler, 1983). Antimicrobial peptides (AMPs) are bioactive molecules that can kill bacteria by disrupting their cellular membrane structure, thus, few scientists tried the AMPs to stop the growth of fungi also. For the management of mycotoxin-producing *Aspergillus*, particularly *A. flavus*, several synthetic peptides, including D4E1, MSI99, and AGM182, have been described by De Lucca et al. (1998), DeGray et al. (2001), Rajasekaran et al. (2001, 2018). Yan et al. (2004) showed that *Achromobacter xylosoxidans* secreted cyclic dipeptide cyclo(L-leucyl-L-prolyl) along with cyclo(D-leucyl-D-prolyl) and cyclo(L-prolyl-Lvalyl) inhibited the aflatoxin production from *Aspergillus parasiticus*. Inoguchi et al. (2019) reported that both blasticidin A inhibited the aflatoxin biosynthesis which acts as protein tyrosine phosphatase inhibitors which in turn can perform as potential aflatoxin production inhibitors.

## Conclusion

Numerous natural substances affect the aflatoxin contamination in food/feeds in a variety of ways, including by suppressing the formation of aflatoxigenic fungus, preventing the biosynthesis of aflatoxin, and removing or degrading aflatoxin. These inhibitors have the potential to replace or supplement existing ways of combating aflatoxin contamination in food and are very promising in this regard. Aflatoxin biosynthesis is selectively targeted by several inhibitors of aflatoxin formation, which do not influence the growth of the fungus cells. However, at greater concentrations, most of the inhibitors also prevent the growth of fungi. This would mean that the secondary metabolism (aflatoxin) is vulnerable to stress brought on by low levels of chemicals that restrict growth. Natural inhibitors are being discovered at an increasing rate, however, most of them still have unclear mechanisms of action. More study is needed to fully comprehend the mechanisms of action of such chemicals before they can be employed commercially on a large scale. Researchers are currently merging datasets from the profiling of transcripts, proteins, and metabolites produced by inhibitory agents with various mechanisms of action to better understand the various aspects of aflatoxin control.

## Author contributions

MMA drafted and wrote half of the manuscript. FQ wrote rest half of the manuscript. MS helped in literature search and editing. MZA designed and did the proofreading. All authors contributed to the article and approved the submitted version.

## Conflict of interest

The authors declare that the research was conducted in the absence of any commercial or financial relationships that could be construed as a potential conflict of interest.

## Publisher’s note

All claims expressed in this article are solely those of the authors and do not necessarily represent those of their affiliated organizations, or those of the publisher, the editors and the reviewers. Any product that may be evaluated in this article, or claim that may be made by its manufacturer, is not guaranteed or endorsed by the publisher.

## References

[ref1] Abdin M. Z. Ahmad M. M. Javed S. (2010). Advances in molecular detection of *Aspergillus*: an update. Arch. Microbiol. 192, 409–425. doi: 10.1007/s00203-010-0563-y, PMID: 20358179

[ref2] Adye J. Mateles R. I. (1964). Incorporation of labeled compounds into aflatoxins. Biochim. Biophys. Acta 86, 418–420. doi: 10.1016/0304-4165(64)90077-7, PMID: 14171025

[ref3] Ahlberg S. Joutsjoki V. Laurikkala S. Varmanen P. Korhonen H. (2017). *Aspergillus flavus* growth inhibition by *lactobacillus* strains isolated from traditional fermented Kenyan milk and maize products. Arch. Microbiol. 199, 457–464. doi: 10.1007/s00203-016-1316-3, PMID: 27816987

[ref4] Akocak P. B. Churey J. J. Worobo R. W. (2015). Antagonistic effect of chitinolytic pseudomonas and bacillus on the growth of fungal hyphae and spores of aflatoxigenic *Aspergillus flavus*. Food Biosci. 10, 48–58. doi: 10.1016/j.fbio.2015.01.005

[ref5] Baiano A. del Nobile M. A. (2015). Antioxidant compounds from vegetable matrices: biosynthesis, occurrence, and extraction systems. Crit. Rev. Food Sci. Nutr. 56, 2053–2068. doi: 10.1080/10408398.2013.812059 25751787

[ref7] Bhatnagar D. McCormick S. P. (1988). The inhibitory effect of neem (*Azadirachta indica*) leaf extracts on aflatoxin synthesis in *Aspergillus parasiticus*. J. Am. Oil Chem. Soc. 65, 1166–1168. doi: 10.1007/BF02660575

[ref8] Bhatnagar D. Rajasekaran K. Payne G. A. R. Y. Brown R. Yu J. Cleveland T.. (2008). The 'omics' tools: genomics, proteomics, metabolomics and their potential for solving the aflatoxin contamination problem. World Mycotoxin J. 1, 3–12. doi: 10.3920/WMJ2008.x001

[ref9] Bluma R. V. Etcheverry M. G. (2008). Application of essential oils in maize grain: impact on *Aspergillus* section Flavi growth parameters and aflatoxin accumulation. Food Microbiol. 25, 324–334. doi: 10.1016/j.fm.2007.10.004, PMID: 18206775

[ref10] Bradley P. R. (1992). British herbal compendium, volume 1: A handbook of scientific information on widely used plant drugs, companion to volume 1 of the British herbal pharmacopoeia, British herbal medicine association. 239.

[ref11] Brown R. L. Chen Z. Y. Warburton M. Luo M. Menkir A. Fakhoury A.. (2010). Discovery and characterization of proteins associated with aflatoxin-resistance: evaluating their potential as breeding markers. Toxins 2, 919–933. doi: 10.3390/toxins2040919, PMID: 22069617 PMC3153200

[ref12] Bryden W. L. (1991). “Occurrence and biological effects of cyclopiazonic acid.” in Emerging food safety problems resulting from microbial contamination: Proceedings of the 7th International Symp. of Toxic Microorganisms. eds. Mise K. Richard J. L.; November 12–14, 1991; Tokyo, Japan, 127–147.

[ref13] Buchanan R. L. Lewis D. F. (1984). Caffeine inhibition of aflatoxin synthesis: probable site of action. Appl. Environ. Microbiol. 47, 1216–1220. doi: 10.1128/aem.47.6.1216-1220.1984, PMID: 6331311 PMC240199

[ref14] Cardwell K. F. Cotty P. J. (2002). Distribution of *Aspergillus* section Flavi among field soils from the four agroecological zones of the Republic of Benin. West Africa. Plant Dis. 86, 434–439. doi: 10.1094/PDIS.2002.86.4.434, PMID: 30818721

[ref15] Chen Z. Y. Brown R. L. Lax A. R. Guo B. Z. Cleveland T. E. Russin J. S. (1998). Resistance to *Aspergillus flavus* in corn kernels is associated with a 14-kDa protein. Phytopathology 88, 276–281. doi: 10.1094/PHYTO.1998.88.4.276, PMID: 18944949

[ref16] Crowden R. K. Harborne J. B. Heywood V. H. (1969). Chemosystematics of the Umbelliferae—a general survey. Phytochemistry 8, 1963–1984. doi: 10.1016/S0031-9422(00)88084-X

[ref17] da Cruz Cabral L. Pinto V. F. Patriarca A. (2013). Application of plant derived compounds to control fungal spoilage and mycotoxin production in foods. Int. J. Food Microbiol. 166, 1–14. doi: 10.1016/j.ijfoodmicro.2013.05.026, PMID: 23816820

[ref18] Dai J. Mumper R. J. (2010). Plant phenolics: extraction, analysis and their antioxidant and anticancer properties. Molecules 15, 7313–7352. doi: 10.3390/molecules15107313, PMID: 20966876 PMC6259146

[ref19] Darsanaki R. Miri M. (2013). Aflatoxin M1 contamination in dairy products. J. Sci. Today's World 2, 500–514.

[ref20] De Lucca A. J. Bland J. M. Grimm C. Jacks T. J. Cary J. W. Jaynes J. M.. (1998). Fungicidal properties, sterol binding, and proteolytic resistance of the synthetic peptide D4E1. Can. J. Microbiol. 44, 514–520. doi: 10.1139/w98-032, PMID: 9734302

[ref21] DeGray G. Rajasekaran K. Smith F. Sanford J. Daniell H. (2001). Expression of an antimicrobial peptide via the chloroplast genome to control phytopathogenic bacteria and fungi. Plant Physiol. 127, 852–862. doi: 10.1104/pp.010233, PMID: 11706168 PMC129257

[ref22] Deshpande B. S. Ambedkar S. S. Shewale J. G. (1988). Biologically active secondary metabolites from Streptomyces. Enzym. Microb. Technol. 10, 455–473. doi: 10.1016/0141-0229(88)90023-3

[ref23] Fakhoury A. M. Woloshuk C. P. (1999). Amy1, the α-amylase gene of *Aspergillus flavus*: involvement in aflatoxin biosynthesis in maize kernels. Phytopathology 89, 908–914. doi: 10.1094/PHYTO.1999.89.10.908, PMID: 18944734

[ref24] FDA (Food and Drug Administration) (2011). “U.S. Food and Drug Administration (FDA) mycotoxin regulatory guidance,” in Food and Drug Administration (FDA) U. S. (Washington, D.C.: National Grain and Feed Association).

[ref25] Fukunaga K. Misato T. Ishii I. Asakawa M. (1955). Blasticidin, a new anti-phytopathogenic fungal substance. J. Agric. Chemi. Soc. Japan 19, 181–188. doi: 10.1080/03758397.1955.10857286

[ref26] Geiser D. M. Dorner J. W. Horn B. W. Taylor J. W. (2000). The phylogenetics of mycotoxin and sclerotium production in *Aspergillus flavus* and *Aspergillus oryzae*. Fungal Genet. Biol. 31, 169–179. doi: 10.1006/fgbi.2000.1215, PMID: 11273679

[ref27] Ghanbari R. Aghaee E. M. Rezaie S. Khaniki G. J. Alimohammadi M. Soleimani M.. (2018). The inhibitory effect of lactic acid bacteria on aflatoxin production and expression of aflR gene in *Aspergillus parasiticus*. J. Food Saf. 38:e12413. doi: 10.1111/jfs.12413

[ref28] Gomaa O. M. El Nour S. A. (2014). Aflatoxin inhibition in *Aspergillus flavus* for bioremediation purposes. Ann. Microbiol. 64, 975–982. doi: 10.1007/s13213-013-0732-8

[ref29] Greene-McDowelle D. M. Ingber B. Wright M. S. Zeringue H. J. Jr. Bhatnagar D. Cleveland T. E. (1999). The effects of selected cotton-leaf volatiles on growth, development and aflatoxin production of *Aspergillus parasiticus*. Toxicon 37, 883–893. doi: 10.1016/S0041-0101(98)00209-8, PMID: 10340828

[ref30] Hammond T. M. Andrewski M. D. Roossinck M. J. Keller N. P. (2008). *Aspergillus* mycoviruses are targets and suppressors of RNA silencing. Eukaryot. Cell 7, 350–357. doi: 10.1128/EC.00356-07, PMID: 18065651 PMC2238147

[ref31] Hua S. S. T. Baker J. L. Flores-Espiritu M. (1999). Interactions of saprophytic yeasts with a nor mutant of *Aspergillus flavus*. Appl. Environ. Microbiol. 65, 2738–2740. doi: 10.1128/AEM.65.6.2738-2740.1999, PMID: 10347069 PMC91404

[ref32] Hua S. S. Grosjean O. K. Baker J. L. (1999). Inhibition of aflatoxin biosynthesis by phenolic compounds. Lett. Appl. Microbiol. 29, 289–291. doi: 10.1046/j.1472-765X.1999.00635.x, PMID: 10664967

[ref33] Inoguchi H. Furukawa T. Yoshinari T. Sakuda S. (2019). Inhibition of aflatoxin production by protein tyrosine phosphatase inhibitors, blasticidin a and dephostatin. JSM Mycotoxins 69, 71–79. doi: 10.2520/myco.69-2-6

[ref35] Kim J. H. Campbell B. C. Mahoney N. Chan K. L. Molyneux R. J. (2011). Chemosensitization of aflatoxigenic fungi to antimycin a and strobilurin using salicylaldehyde, a volatile natural compound targeting cellular antioxidation system. Mycopathologia 171, 291–298. doi: 10.1007/s11046-010-9356-8, PMID: 20803256

[ref36] Kim J. H. Campbell B. C. Yu J. Mahoney N. Chan K. L. Molyneux R. J.. (2005). Examination of fungal stress response genes using *Saccharomyces cerevisiae* as a model system: targeting genes affecting aflatoxin biosynthesis by *Aspergillus flavus* link. Appl. Microbiol. Biotechnol. 67, 807–815. doi: 10.1007/s00253-004-1821-1, PMID: 15614562

[ref37] Kim J. H. Mahoney N. Chan K. L. Molyneux R. J. Campbell B. C. (2006). Controlling food-contaminating fungi by targeting their antioxidative stress-response system with natural phenolic compounds. Appl. Microbiol. Biotechnol. 70, 735–739. doi: 10.1007/s00253-005-0123-6, PMID: 16463173

[ref38] Kim Y. H. Woloshuk C. P. Cho E. H. Bae J. M. Song Y. S. Huh G. H. (2007). Cloning and functional expression of the gene encoding an inhibitor against *Aspergillus flavus* α-amylase, a novel seed lectin from *Lablab purpureus* (*Dolichos lablab*). Plant Cell Rep. 26, 395–405. doi: 10.1007/s00299-006-0250-2, PMID: 17149640

[ref39] Kim J. H. Yu J. Mahoney N. Chan K. L. Molyneux R. J. Varga J.. (2008). Elucidation of the functional genomics of antioxidant-based inhibition of aflatoxin biosynthesis. Int. J. Food Microbiol. 122, 49–60. doi: 10.1016/j.ijfoodmicro.2007.11.058, PMID: 18166238

[ref40] Kimura N. Hirano S. (1988). Inhibitory strains of *Bacillus subtilis* for growth and aflatoxin-production of aflatoxigenic fungi. Agric. Biol. Chem. 52, 1173–1179. doi: 10.1271/bbb1961.52.1173

[ref41] Kondo T. Sakurada M. Okamoto S. Ono M. Tsukigi H. Suzuki A.. (2001). Effects of aflastatin a, an inhibitor of aflatoxin production, on aflatoxin biosynthetic pathway and glucose metabolism in *Aspergillus parasiticus*. J. Antibiot. 54, 650–657. doi: 10.7164/antibiotics.54.650, PMID: 11592501

[ref42] Kong Q. Chi C. Yu J. Shan S. Li Q. Li Q.. (2014). The inhibitory effect of *bacillus megaterium* on aflatoxin and cyclopiazonic acid biosynthetic pathway gene expression in *Aspergillus flavus*. Appl. Microbiol. Biotechnol. 98, 5161–5172. doi: 10.1007/s00253-014-5632-8, PMID: 24652062

[ref43] Kong C. Hu F. Xu X. Liang W. Zhang C. (2004). Allelopathic plants. *Ageratum conyzoides* L. Allelopath. J 14, 1–12.

[ref44] Korkina L. G. (2007). Phenylpropanoids as naturally occurring antioxidants: from plant defense to human health. Cell. Mol. Biol. 53, 15–25. doi: 10.1170/T772 17519109

[ref45] Kumar A. Pathak H. Bhadauria S. Sudan J. (2021). Aflatoxin contamination in food crops: causes, detection, and management: a review. Food Prod. Proc. Nutr. 3:17. doi: 10.1186/s43014-021-00064-y

[ref46] Lee S. E. Campbell B. C. Molyneux R. J. Hasegawa S. Lee H. S. (2001). Inhibitory effects of naturally occurring compounds on aflatoxin B1 biotransformation. J. Agric. Food Chem. 49, 5171–5177. doi: 10.1021/jf010454v, PMID: 11714299

[ref47] Lee J. W. Roze L. V. Linz J. E. (2007). Evidence that a wortmannin-sensitive signal transduction pathway regulates aflatoxin biosynthesis. Mycologia 99, 562–568. doi: 10.1080/15572536.2007.11832550, PMID: 18065007

[ref49] Liang X. Q. Luo M. Guo B. Z. (2006). Resistance mechanisms to *Aspergillus flavus* infection and aflatoxin contamination in peanut (*Arachis hypogaea*). Plant Pathol. J. 5, 115–124. doi: 10.3923/ppj.2006.115.124

[ref50] MacRae W. D. Towers G. N. (1984). Biological activities of lignans. Phytochemistry 23, 1207–1220. doi: 10.1016/S0031-9422(00)80428-8

[ref51] Mahoney N. Molyneux R. J. Campbell B. C. (2000). Regulation of aflatoxin production by naphthoquinones of walnut (*Juglans regia*). J. Agric. Food Chem. 48, 4418–4421. doi: 10.1021/jf0003449, PMID: 10995372

[ref52] Mannaa M. Oh J. Kim K. (2017). Microbe-mediated control of *Aspergillus flavus* in stored rice grains with a focus on aflatoxin inhibition and biodegradation. Ann. Appl. Biol. 171, 376–392. doi: 10.1111/aab.12381

[ref53] Miller J. D. Fielder D. A. Dowd P. F. Norton R. A. Collins F. W. (1996). Isolation of 4-acetyl-benzoxazolin-2-one (4-ABOA) and diferuloylputrescine from an extract of *Gibberella* ear rot-resistant corn that blocks mycotoxin biosynthesis, and the insect toxicity of 4-ABOA and related compounds. Biochem. Syst. Ecol. 24, 647–658. doi: 10.1016/S0305-1978(96)00050-6

[ref54] Misaghi I. J. Cotty P. J. Decianne D. M. (1995). Bacterial antagonists of *Aspergillus flavus*. Biocontrol Sci. Tech. 5, 387–392. doi: 10.1080/09583159550039846

[ref55] Nerilo S. B. Romoli J. C. Z. Nakasugi L. P. Zampieri N. S. Mossini S. A. G. Rocha G. H. O.. (2020). Antifungal activity and inhibition of aflatoxins production by *Zingiber officinale* roscoe essential oil against *Aspergillus flavus* in stored maize grains. Ciência Rural 50:e20190779. doi: 10.1590/0103-8478cr20190779

[ref56] Newall C. A. Anderson L. A. Phillipson J. D. (1996). Herbal medicines: A guide for healthcare professionals. 2nd Edn.London: Pharmaceutical Press, 432.

[ref57] Nogueira J. H. Gonçalez E. Galleti S. R. Facanali R. Marques M. O. Felício J. D. (2010). *Ageratum conyzoides* essential oil as aflatoxin suppressor of *Aspergillus flavus*. Int. J. Food Microbiol. 137, 55–60. doi: 10.1016/j.ijfoodmicro.2009.10.017, PMID: 19906457

[ref58] Norton R. A. (1997). Effect of carotenoids on aflatoxin B1 synthesis by *Aspergillus flavus*. Phytopathology 87, 814–821. doi: 10.1094/PHYTO.1997.87.8.814, PMID: 18945049

[ref59] Norton R. A. (1999). Inhibition of aflatoxin B1 biosynthesis in *Aspergillus flavus* by anthocyanidins and related flavonoids. J. Agric. Food Chem. 47, 1230–1235. doi: 10.1021/jf980995t, PMID: 10552442

[ref60] Ono M. Sakuda S. Suzuki A. Isogai A. (1997). Aflastatin a, a novel inhibitor of aflatoxin production by aflatoxigenic fungi. J. Antibiot. 50, 111–118. doi: 10.7164/antibiotics.50.111, PMID: 9099219

[ref61] Passone M. A. Girardi N. S. Etcheverry M. (2013). Antifungal and antiaflatoxigenic activity by vapor contact of three essential oils, and effects of environmental factors on their efficacy. LWT-Food Sci. Technol. 53, 434–444. doi: 10.1016/j.lwt.2013.03.012

[ref62] Payne G. A. Hagler W. M. Jr. (1983). Effect of specific amino acids on growth and aflatoxin production by *Aspergillus parasiticus* and *Aspergillus flavus* in defined media. Appl. Environ. Microbiol. 46, 805–812. doi: 10.1128/aem.46.4.805-812.1983, PMID: 6416168 PMC239471

[ref63] Pereyra M. L. G. Martínez M. P. Petroselli G. Balsells R. E. Cavaglieri L. R. (2018). Antifungal and aflatoxin-reducing activity of extracellular compounds produced by soil *bacillus* strains with potential application in agriculture. Food Control 85, 392–399. doi: 10.1016/j.foodcont.2017.10.020

[ref64] Prakash B. Kedia A. Mishra P. K. Dwivedy A. K. Dubey N. K. (2015). Assessment of chemically characterized *Rosmarinus officinalis* L. essential oil and its major compounds as plant-based preservative in food system based on their efficacy against food-borne moulds and aflatoxin secretion and as antioxidant. Int. J. Food Sci. Technol. 50, 1792–1798. doi: 10.1111/ijfs.12822

[ref65] Rajasekaran K. Cary J. W. Cleveland T. E. (2006). Prevention of preharvest aflatoxin contamination through genetic engineering of crops. Mycotox. Res. 22, 118–124. doi: 10.1007/BF02956775, PMID: 23605584

[ref66] Rajasekaran K. Sayler R. J. Sickler C. M. Majumdar R. Jaynes J. M. Cary J. W. (2018). Control of *Aspergillus flavus* growth and aflatoxin production in transgenic maize kernels expressing a tachyplesin-derived synthetic peptide, AGM182. Plant Sci. Int. J. Exp. Plant Biol. 270, 150–156. doi: 10.1016/j.plantsci.2018.02.006, PMID: 29576068

[ref67] Rajasekaran K. Stromberg K. D. Cary J. W. Cleveland T. E. (2001). Broad-spectrum antimicrobial activity *in vitro* of the synthetic peptide D4E1. J. Agric. Food Chem. 49, 2799–2803. doi: 10.1021/jf010154d, PMID: 11409968

[ref68] Razzaghi-Abyaneh M. Allameh A. Tiraihi T. Shams-Ghahfarokhi M. Ghorbanian M. (2005). Morphological alterations in toxigenic *Aspergillus parasiticus* exposed to neem (*Azadirachta indica*) leaf and seed aqueous extracts. Mycopathologia 159, 565–570. doi: 10.1007/s11046-005-4332-4, PMID: 15983743

[ref69] Razzaghi-Abyaneh M. Shams-Ghahfarokhi M. Chang P.-K. (2011). “Aflatoxins: mechanisms of inhibition by antagonistic plants and microorganisms,” in Aflatoxins - biochemistry and molecular biology. ed. Guevara-González R. G. (London: InTechOpen).

[ref70] Razzaghi-Abyaneh M. Shams-Ghahfarokhi M. Rezaee M. B. Sakuda S. (2009). “Natural aflatoxin inhibitors from medicinal plants,” in Mycotoxins in food, feed and bioweapons. eds. Rai M. Varma A. (Berlin, Heidelberg: Springer), 329–352.

[ref71] Razzaghi-Abyaneh M. Shams-Ghahfarokhi M. Yoshinari T. Rezaee M. B. Jaimand K. Nagasawa H.. (2008). Inhibitory effects of *Satureja hortensis* L. essential oil on growth and aflatoxin production by *Aspergillus parasiticus*. Int. J. Food Microbiol. 123, 228–233. doi: 10.1016/j.ijfoodmicro.2008.02.003, PMID: 18353477

[ref72] Razzaghi-Abyaneh M. Yoshinari T. Shams-Ghahfarokhi M. Rezaee M. B. Nagasawa H. Sakuda S. (2007). Dillapiol and apiol as specific inhibitors of the biosynthesis of aflatoxin G1 in *Aspergillus parasiticus*. Biosci. Biotechnol. Biochem. 71, 2329–2332. doi: 10.1271/bbb.70264, PMID: 17827697

[ref73] Ren Y. Jin J. Zheng M. Yang Q. Xing F. (2020). Ethanol inhibits aflatoxin B1 biosynthesis in *Aspergillus flavus* by up-regulating oxidative stress-related genes. Front. Microbiol. 10:2946. doi: 10.3389/fmicb.2019.02946, PMID: 32010073 PMC6978751

[ref74] Ren X. Zhang Q. Zhang W. Mao J. Li P. (2020). Control of Aflatoxigenic molds by antagonistic microorganisms: inhibitory behaviors, bioactive compounds, related mechanisms, and influencing factors. Toxins 12:24. doi: 10.3390/toxins12010024, PMID: 31906282 PMC7020460

[ref75] Reverberi M. Fabbri A. A. Zjalic S. Ricelli A. Punelli F. Fanelli C. (2005). Antioxidant enzymes stimulation in *Aspergillus parasiticus* by *Lentinula edodes* inhibits aflatoxin production. Appl. Microbiol. Biotechnol. 69, 207–215. doi: 10.1007/s00253-005-1979-1, PMID: 15838675

[ref76] Rondinone C. M. Carvalho E. Rahn T. Manganiello V. C. Degerman E. Smith U. P. (2000). Phosphorylation of PDE3B by phosphatidylinositol 3-kinase associated with the insulin receptor. J. Biol. Chem. 275, 10093–10098. doi: 10.1074/jbc.275.14.10093, PMID: 10744689

[ref77] Sakuda S. Ono M. Ikeda H. Nakamura T. Inagaki Y. Kawachi R.. (2000). Blasticidin a as an inhibitor of aflatoxin production by *Aspergillus parasiticus*. J. Antibiot. 53, 1265–1271. doi: 10.7164/antibiotics.53.1265, PMID: 11213287

[ref78] Salamon I. (1992). Chamomile: a medicinal plant. Herb. Spice Med. Plant Dig. U. S/ A. 1, 1–5.

[ref79] Schmidt F. R. (2009). The RNA interference—virus interplay: tools of nature for gene modulation, morphogenesis, evolution and a possible mean for aflatoxin control. Appl. Microbiol. Biotechnol. 83, 611–615. doi: 10.1007/s00253-009-2007-7, PMID: 19466405

[ref80] Shu X. Wang Y. Zhou Q. Li M. Hu H. Ma Y.. (2018). Biological degradation of aflatoxin B1 by cell-free extracts of bacillus velezensis DY3108 with broad PH stability and excellent thermostability. Toxins 10:330. doi: 10.3390/toxins10080330, PMID: 30110983 PMC6116002

[ref81] Siahmoshteh F. Hamidi-Esfahani Z. Spadaro D. Shams-Ghahfarokhi M. Razzaghi-Abyaneh M. (2018). Unraveling the mode of antifungal action of *Bacillus subtilis* and bacillus amyloliquefaciens as potential biocontrol agents against aflatoxigenic *Aspergillus parasiticus*. Food Control 89, 300–307. [CrossRef]. doi: 10.1016/j.foodcont.2017.11.010

[ref82] Sonker N. Pandey A. K. Singh P. (2015). Efficiency of *Artemisia nilagirica* (Clarke) Pamp. Essential oil as a mycotoxicant against postharvest mycobiota of table grapes. J. Sci. Food Agric. 95, 1932–1939. doi: 10.1002/jsfa.6901, PMID: 25199920

[ref83] Takeuchi T. Aoyanagi T. Okami Y. Osanawa H. Iinuma H. Ogawa K. (1991). Novel aminopeptidase-inhibiting dioctatins and their manufacture with *Streptomyces* species. Jap. Pat. 3077857:A2.

[ref84] Thanaboripat D. Mongkontanawut N. Suvathi Y. Ruangrattanamatee V. (2004). Inhibition of aflatoxin production and growth of *Aspergillus flavus* by citronella oil. Cur. Appl. Sci. Technol. 4, 1–8. doi: 10.1170/T772

[ref85] Thippeswamy S. Mohana D. C. Abhishek R. U. Manjunath K. (2014). Inhibitory activity of plant extracts on Aflatoxin B1 biosynthesis by *Aspergillus flavus*. J. Agric. Sci. Technol. 16, 1123–1132.

[ref86] Tolouee M. Alinezhad S. Saberi R. Eslamifar A. Zad S. J. Jaimand K.. (2010). Effect of *Matricaria chamomilla* L. flower essential oil on the growth and ultrastructure of *Aspergillus Niger* van Tieghem. Int. J. Food Microbiol. 139, 127–133. doi: 10.1016/j.ijfoodmicro.2010.03.032, PMID: 20385420

[ref88] Verheecke C. Liboz T. Anson P. Diaz R. Mathieu F. (2015). Reduction of aflatoxin production by *Aspergillus flavus* and *Aspergillus parasiticus* in interaction with *Streptomyces*. Microbiology 161, 967–972. doi: 10.1099/mic.0.000070, PMID: 25741015

[ref89] Wicklow D. T. Norton R. A. McAIpin C. E. (1998). β-Carotene inhibition of aflatoxin biosynthesis among *Aspergillus flavus* genotypes from Illinois corn. Mycoscience 39, 167–172. doi: 10.1007/BF02464055

[ref90] Wright M. S. Greene-McDowelle D. M. Zeringue H. J. Jr. Bhatnagar D. Cleveland T. E. (2000). Effects of volatile aldehydes from *Aspergillus*-resistant varieties of corn on *Aspergillus parasiticus* growth and aflatoxin biosynthesis. Toxicon 38, 1215–1223. doi: 10.1016/S0041-0101(99)00221-4, PMID: 10736475

[ref91] Yan P. S. Song Y. Sakuno E. Nakajima H. Nakagawa H. Yabe K. (2004). Cyclo (L-leucyl-L-prolyl) produced by *Achromobacter xylosoxidans* inhibits aflatoxin production by *Aspergillus parasiticus*. Appl. Environ. Microbiol. 70, 7466–7473. doi: 10.1128/AEM.70.12.7466-7473.2004, PMID: 15574949 PMC535151

[ref92] Yang X. Zhang Q. Chen Z. Y. Liu H. Li P. (2017). Investigation of *Pseudomonas fluorescens* strain 3JW1 on preventing and reducing aflatoxin contaminations in peanuts. PLoS One 12:e0178810. doi: 10.1371/journal.pone.0178810, PMID: 28640833 PMC5480873

[ref93] Yoshinari T. Akiyama T. Nakamura K. Kondo T. Takahashi Y. Muraoka Y.. (2007). Dioctatin a is a strong inhibitor of aflatoxin production by *Aspergillus parasiticus*. Microbiology 153, 2774–2780. doi: 10.1099/mic.0.2006/005629-0, PMID: 17660441

[ref94] Yoshinari T. Noda Y. Yoda K. Sezaki H. Nagasawa H. Sakuda S. (2010). Inhibitory activity of blasticidin a, a strong aflatoxin production inhibitor, on protein synthesis of yeast: selective inhibition of aflatoxin production by protein synthesis inhibitors. J. Antibiot. 63, 309–314. doi: 10.1038/ja.2010.36, PMID: 20414321

[ref95] Yoshinari T. Yaguchi A. Takahashi-Ando N. Kimura M. Takahashi H. Nakajima T.. (2008). Spiroethers of German chamomile inhibit production of aflatoxin G1 and trichothecene mycotoxin by inhibiting cytochrome P450 monooxygenases involved in their biosynthesis. FEMS Microbiol. Lett. 284, 184–190. doi: 10.1111/j.1574-6968.2008.01195.x, PMID: 18492060

[ref96] Zeringue H. J. Brown R. L. Neucere J. N. Cleveland T. E. (1996). Relationships between C6−C12 alkanal and alkenal volatile contents and resistance of maize genotypes to *Aspergillus flavus* and aflatoxin production. J. Agric. Food Chem. 44, 403–407. doi: 10.1021/jf950313r

[ref97] Zeringue H. J. Jr. (1992). Effects of C6-C10 alkenals and alkanals on eliciting a defence response in the developing cotton boll. Phytochemistry 31, 2305–2308. doi: 10.1016/0031-9422(92)83269-5

[ref98] Zhao X. Zhi Q. Q. Li J. Y. Keller N. P. He Z. M. (2018). The antioxidant gallic acid inhibits aflatoxin formation in *Aspergillus flavus* by modulating transcription factors FarB and CreA. Toxins 10:270. doi: 10.3390/toxins10070270, PMID: 29970790 PMC6071284

[ref99] Zhou W. Hu L. Zhao Y. Wang M. Zhang H. Mo H. (2015). Inhibition of fungal aflatoxin B1 biosynthesis by diverse botanically derived polyphenols. Trop. J. Pharm. Res. 14, 605–609. doi: 10.4314/tjpr.v14i4.7

[ref100] Zjalic S. Reverberi M. Ricelli A. Granito V. M. Fanelli C. Fabbri A. A. (2006). *Trametes versicolor*: a possible tool for aflatoxin control. Int. J. Food Microbiol. 107, 243–249. doi: 10.1016/j.ijfoodmicro.2005.10.003, PMID: 16337299

